# Bio-functionalized nanocolloids of ZnS quantum dot/amine-rich polypeptides for bioimaging cancer cells with antibacterial activity: “*seeing is believing*”†

**DOI:** 10.1039/d3ra06711d

**Published:** 2023-11-23

**Authors:** Alexandra A. P. Mansur, Sandhra C. Carvalho, Elaine M. S. Dorneles, Andrey P. Lage, Zelia I. P. Lobato, Herman S. Mansur

**Affiliations:** a Department of Metallurgical and Materials Engineering, Center of Nanoscience, Nanotechnology, and Innovation-CeNano^2^I, Federal University of Minas Gerais, UFMG Av. Antônio Carlos, 6627 – Escola de Engenharia, Bloco 2 – Sala 2233 31.270-901 Belo Horizonte MG Brazil hmansur@demet.ufmg.br +55-31-34091843 +55-31-34091843; b Departamento de Medicina Veterinária, Universidade Federal de Lavras, UFLA Brazil; c Departamento de Medicina Veterinária Preventiva, Federal University of Minas Gerais, UFMG Brazil

## Abstract

Among almost 200 types of cancers, glioma is considered one of the most common forms of malignant tumors located in the central nervous system (CNS). Glioblastoma (GBM), one of the deadliest types of brain cancer, remains one of the challenges faced by oncologists. Thus, smartly designed nanomaterials biofunctionalized with polypeptides can offer disruptive strategies relying on the earliest possible diagnosis (“*seeing is believing*”) combined with more efficient therapies for fighting cancer cells. To worsen this scenario, bacteria infections very often pose a serious challenge to cancer-immunodeficient patients under chemotherapy. Thus, in this research, we report for the first time the design and synthesis of novel nanoconjugates composed of photoluminescent ZnS quantum dots (ZnS QDs), which were directly surface biofunctionalized with epsilon-poly-l-lysine (εPL), acting as an amine-rich cell-penetrating peptide (CPP) and antimicrobial peptide agent (AMP). These nanoconjugates (named ZnS@CPP–AMP) were produced through a one-step facile, eco-friendly, and biocompatible colloidal aqueous process to be applied as a proof of concept as nanoprobes for bioimaging GBM cancer cells (U87-MG) associated with synergic antibacterial activity. They were characterized regarding their physicochemical and optical properties associated with the biological activity. The results demonstrated that chemically stable aqueous colloidal nanoconjugates were effectively formed, resembling core–shell (inorganic, ZnS, organic, εPL) nanostructures with positively surface-charged features due to the cationic nature of the amine-rich polypeptide. More importantly, they demonstrated photoluminescent activity, cytocompatibility *in vitro*, and no significant intracellular reactive oxygen species (ROS) generation. These ZnS@CPP–AMP nanocolloids behaved as fluorescent nanoprobes for bioimaging GBM cancer cells, where the polycationic nature of the εPL biomolecule may have enhanced the cellular uptake. Additionally, they displayed mild antibacterial growth inhibition due to electrostatic interactions with bacterial membranes. Thus, it can be envisioned that these novel photoluminescent colloidal nanoconjugates offer novel nanoplatforms that can be specifically targeted with biomolecules for bioimaging to diagnose highly lethal cancers, such as GBM, and as an adjuvant in antibacterial therapy.

## Abstract 2

Nanotechnology meets oncology: peptide-functionalized nanohybrids for bioimaging cancer cells and antibacterial activity.

## Graphical abstract

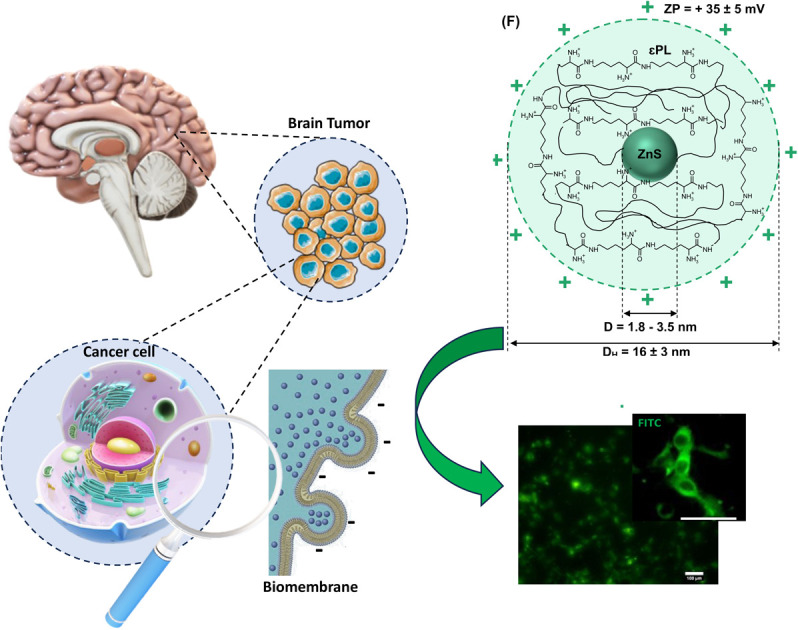

## 1. Introduction

Oncotherapy has experienced noteworthy progress in recent decades, although cancer remains one of the most lethal diseases of the current century, second only to cardiovascular illnesses. Among almost 200 types of cancers, glioma is considered one of the most common forms of malignant tumors located in the central nervous system (CNS). It encompasses almost 80% of primary malignant types of brain tumors. In particular, glioblastoma (GBM) is the most aggressive type of glioma, which accounts for approximately 50% of primary malignant brain tumors.^1–3^ Unfortunately, this scenario worsens because few possibilities of treatment are currently available for brain cancers. Thus, to overcome the hurdles faced in cancer diagnosis and therapy, nano-oncology relies on a new strategy of merging the fields of nanotechnology, materials science, medicine, immunology, and biology, termed nanomedicine.^4^ As a result, a sophisticated generation of novel hybrid nanomaterials has recently emerged focused on nanomedicine for fighting cancer. The nanohybrids encompass the key characteristics of functional polymers and biomolecules with the properties of inorganic nanoparticles, creating a myriad of new nanomaterials.^3,5,6^ They ultimately aim to drastically transform cancer research and clinical practice by providing innovative nanotools not limited to expanding the current diagnostic and therapeutic alternatives but also creating disruptive strategies to surpass the numerous challenges faced by oncology.^5,6^ They amalgamate versatile physicochemical properties and structural features of polymer macromolecules (“soft matter”) with multiple characteristics of inorganic nanoparticles (“hard matter”), such as optical, magnetic, and electronic. As a result, their integration permits the construction of designed nanostructures and supramolecular nanoassemblies, forming exciting hybrid nanomaterials. These nanohybrids combine the functions of each component separately to ascribe new additional properties and features through synergic effects.^5^

Hence, the development of hybrid nanomaterials has boomed in recent years, where polymer-based systems have been engineered with inorganic nanomaterials in diverse combinations to take full advantage of the response in cancer diagnosis and therapy (termed nanotheranostics). As “hard matter” representatives, inorganic nanoparticles have been intensively researched as stimuli-responsive materials for applications in cancer theranostics. They include excitonic nanomaterials, such as semiconductor fluorophores (*e.g.*, quantum dots, QDs), plasmonic nanophotonic materials, such as noble metal nanoparticles (*e.g.*, Au, Ag), and superparamagnetic nanoparticles based on iron-oxide compounds (SPION). They can exhibit outstanding properties, making them unique weapons for fighting against cancer.^3,7^ In this sense, inorganic semiconductor QDs have gained considerable attention from researchers as the preferred choice in nanophotonics as excitonic-based nanomaterials in diagnostics, biomedical imaging, and biosensing. They are noticeable fluorescent semiconductor nanocrystals with emission tunable from visible to infrared wavelengths. These unique features are credited to their size-dependent optical properties, confining the exciton (*i.e.*, coupled electron–hole pair, e^−^/h^+^) within the quantum regime.

QDs are inorganic semiconductor fluorophores with outstanding optoelectronic properties, including a narrow optical emission profile and a high extinction coefficient associated with potential multiple-signal detection.^2^ Therefore, they are highly interesting candidates for cell bioimaging purposes applied in cancer diagnosis. Zinc sulfide quantum dots (ZnS QDs), a wide-bandgap semiconductor (II–IV group), and Zn-bearing nanosized compounds (*e.g.*, binary, ternary, and multinary alloys) have been broadly studied due to their unique photonic characteristics suitable for numerous applications. They encompass from nanophotocatalysis to bioimaging and biosensing nanomaterials for detecting and diagnosing several diseases, where cancer has been subjected to intensive research.^8–14^ Hence, credited to these photoluminescence properties, ZnS-based hybrid nanosystems have been employed as biofunctional cellular nanoprobes for bioimaging. Moreover, by adopting a “green” chemistry strategy in developing innovative nanomedicines, ZnS QDs are considered non-toxic compared to most conventional semiconductor QDs, which are generally synthesized *via* metallorganic routes using highly toxic heavy metals (*e.g.*, Cd^2+^, Pb^2+^). On the contrary, ZnS QDs can be easily synthesized through aqueous colloidal processes using water-soluble polymers and biomolecules under mild conditions.^9,10^ So, besides the unique optical properties for developing cancer nanomedicines, these aspects make ZnS-based QDs very favorable for environmentally benign applications.^2^ Overall, ZnS QDs offer great potential as a non-invasive and highly sensitive nanotool for cancer cell imaging, aiding in early detection and diagnostics while assisting in monitoring cancer progression and optically guided surgery. Nonetheless, because of their extremely small size, like most nanosized materials, these fluorescent nanoprobes are unstable in aqueous media because they possess huge surface energy, tending to grow or agglomerate. Therefore, they must be (bio)chemically stabilized by capping molecules as ligands to form water-dispersible nanocolloids, preserving their unique optoelectronic properties.^15^

Among several “soft matter” alternatives for building hybrid nanomaterials, biopolymers and biomolecules have been the most preferred choice for developing supramolecular colloidal nanostructures. They possess structures that can display dynamic changes with reversibility of shape and conformation in response to applied external stimuli. Thus, these exceptional characteristics turn biopolymers and biomolecules into promising candidates for cancer nanomedicine.^9,16^ Considering the “soft matter” from natural origin, polysaccharides (*e.g.*, cellulose, chitosan, hyaluronic acid, and their semi-processed derivatives) and proteins (*e.g.*, collagen and polypeptides) have often been selected as the best options of biocompatible (macro)molecules for combining with inorganic “hard matter,” rendering nanohybrids for cancer diagnosis and therapy applications.^17^ They can be chemically modified to increase water-solubility and behave simultaneously as capping ligands for stabilizing the inorganic nanomaterial and biofunctional agent, producing colloidal core–shell inorganic–organic hybrid nanostructures. Moreover, these nanosystems can be associated with drugs *via* bioconjugation and complexation, producing supramolecular “vesicle-like” multifunctional nanoarchitectures with excellent properties for cancer targeting, imaging, and drug delivery in cancer research.^9,17,18^ It is important to highlight that, as a pivotal strategy, advanced bioconjugates have been built by coupling (covalently or not) ligands with affinity biomolecules such as antibodies, peptides, folic acid, *etc.*, for developing new cancer nanomedicines for diagnosis and therapy to specifically target and kill cancer cells while the preserving healthy ones. This approach, distinct from unspecific interactions, permits more precise diagnosis and higher efficiency for reaching the tumor site, reducing potential systemic collateral effects frequently observed in cancer patients undergoing chemotherapy.^19^

Therefore, polypeptides separated or combined with biopolymers (often termed bioconjugates) have gained increasing attention as candidates for the next generation of anticancer hybrid nanomedicines.^18,20–22^ Polypeptides can offer several advantages, including fast synthesis and chemical functionalization, and they are less likely to cause immunogenicity than proteins or antibodies (natural or synthetic). Besides, favored by their smaller size, they can better penetrate cells, tissues, and oncogenic regions, helping tumor detection and therapeutic drug delivery. Moreover, owing to their great similarity to protein structure, short polypeptides, compared to other small organic molecules, present greater efficacy, selectivity, and specificity as chemotherapeutic anticancer agents. The polypeptides incorporated into nanohybrids can be more prone to protease enzymatic reaction, usually leading to degradation products with lower systemic toxicity and minimizing tissue accumulation.^20–22^

Specifically, a polycationic polypeptide, epsilon-poly-l-lysine (εPL), has been demonstrated to augment the cellular uptake of nanoparticles. The most accepted mechanism of cellular uptake of poly-lysines, as amine-rich polypeptides, involves endocytosis due to numerous interactions at the nano-bio interfaces, where the peptide biomolecule is engulfed by the cell membrane and transported into the cytoplasmic compartment. Once inside, the nanosystem can be activated by an external stimulus (*i.e.*, optical or magnetic) and also release its cargo, such as drugs, small molecules, proteins, or nucleic acids.^17,23,24^ Thus, the εPL can be applied as cell-penetrating peptides (CPP) by forming a complex at nano-biointerfaces with carbohydrates of the cancer cell surface, targeting drug nanocarriers towards the tumor microenvironment.^17,22–25^

Besides the advantages mentioned above for favoring cellular uptake, because of their cationic characteristics and amphiphilic structures, epsilon-poly-l-lysine has also been widely explored as an antimicrobial peptide (AMP). It has been reported that εPL-based nanosystems present antibacterial activity predominantly ascribed to disrupting, negatively charged bacterial membranes. As a result, they inactivate nucleic acids and cytoplasmic proteins, inhibiting growth or causing bacteria death.^26^ In this view, nanostructures bearing epsilon-poly-l-lysine polypeptides are particularly highly useful in the field of nanomedicine, as they can enhance the more efficient uptake of anti-tumor agents by cancer cells while promoting a barrier against bacterial infections. Thus, εPL polypeptide may be interpreted as a case of synergic effect (*i.e.*, “*one stone, two birds*”), simultaneously amalgamating the function of tumor-penetrating peptide and antibacterial bioactive peptide in one particular sequence.^24,27^ Besides being applied as an adjuvant in cancer research due to its high importance, the antibacterial activity of biomolecules, separated or forming nanocomposites, has been investigated for a myriad of other applications. For instance, they can be essential in preventing bacterial infections in wound healing for skin tissue engineering and as antimicrobial agents in food packing.^28^

Albeit being a captivating and intensively researched theme in recent years, fluorescent “cadmium-free” quantum dots such as ZnS QDs directly stabilized and biologically functionalized by polypeptides using a facile “green” aqueous process for cancer nanomedicine are scarcely reported in the literature. Until now, most cancer nanomedicines have been built through complex bioconjugation chemistry principles with other ligands, macromolecules, and polymers.^9,10,22,29^

Thus, this study reports, for the first time, the design and production of novel nanohybrids composed of an inorganic ZnS quantum dot core (ZnS QD) directly stabilized and biofunctionalized with epsilon-poly-l-lysine (εPL) polypeptide shell, acting as an amine-rich cell-penetrating peptide (CPP) and antimicrobial peptide agent (AMP). They were produced using strictly an environmentally friendly aqueous process, forming stable supramolecular water-dispersed colloidal inorganic–organic nanohybrids (ZnS@CPP–AMP). Moreover, these nanohybrids have been comprehensively characterized to assess their physicochemical and biological properties. As a proof-of-concept, they concurrently demonstrated to be suitable as luminescent nanoprobes for bioimaging brain cancer cells *in vitro*, where the εPL as an amine-rich polypeptide enhanced cellular uptake and presented mild antibacterial activity. Thus, it may be anticipated that, with further studies integrating targeting biomolecules, they hold future potential for applications in bioimaging for cancer diagnosis while as an adjuvant in preventing microbial infections often found in immunodeficient patients submitted to chemotherapy.

## 2. Materials and methods

### 2.1. Materials

Zinc chloride (ZnCl_2_ > 98%) and sodium sulfide nonahydrate (Na_2_S·9H_2_O, >98%) were supplied by Sigma-Aldrich (United States of America, USA). Epsilon-poly-l-lysine hydrochloride (εPL, molar mass, MM, 3500–4500 Da, >98%) was provided by Biosynth Carbosynth Group (United Kingdom, UK). All chemicals were used without further purifications, and deionized water (DI water, Millipore Simplicity™, resistivity > 18 MΩ cm) was used to make the solutions. Unless specified otherwise, the protocols and procedures were executed at room temperature (RT, 25 ± 2 °C).

### 2.2. Synthesis of ZnS@CPP–AMP nanoconjugates

The colloidal nanoconjugates made of ZnS nanoparticles with εPL as capping ligand and biofunctionalization agent were synthesized as briefly summarized here: 2 mL of εPL solution (1% w/v) and 39 mL of DI water were added to the flask reacting vessel (pH ∼4.5). Under moderate magnetic stirring, 6.0 mL of ZnCl_2_ salt solution (10 mM) and 6.0 mL of sulfide precursor solution (Na_2_S·9H_2_O, 10 mM) were added to the flask (Zn^2+^ : S^2−^ molar ratio = 1 : 1) and stirred for 60 min, reaching a final pH = 5.8 ± 0.1. The nanoconjugates were purified by dialysis against distilled water for 24 h using Pur-A-Lyzer™ Maxi Dialysis Kit (3.5 kDa molecular weight cut-off, Sigma-Aldrich, USA) with three changes of dialysis medium. This protocol produced a nicely uniform and clear solution stored (room temperature, dry place, sheltered from light) for further use.

### 2.3. Characterization of ZnS@CPP–AMP colloid

ZnS@CPP–AMP water-dispersed nanoconjugates were extensively characterized by several techniques for assessing their morphological, structural, and spectroscopic features.

Ultraviolet-visible (UV-Vis) spectroscopy analyses of the nanocolloids were executed to assess their optical absorption activity using Lambda EZ-210 in transmission mode (PerkinElmer).

The photoluminescence spectroscopy (PL) of ZnS@CPP–AMP nanomaterials was performed to investigate their optical emission behavior, using FluoroMax-Plus-CP (Horiba Scientific) to obtain steady-state spectra and 3D (three-dimensional) excitation/emission contour mappings.

Dynamic light scattering (DLS) and electrokinetic potential (zeta potential, ZP) characterizations were performed using ZetaPlus instrument (Brookhaven Instruments Corporation), which are considered relevant analytical tools for studying colloidal suspensions.

Fourier-transform infrared spectroscopy (FTIR), an important technique for chemical analysis of molecules and colloids, was performed by the attenuated total reflectance method (ATR, Nicolet 6700, Thermo Fisher). X-ray photoelectron spectroscopy (XPS) analysis, as a surface chemistry technique for the characterization of materials, was used based on Mg-Kα as the excitation source in Amicus equipment (Kratos Analytical).

For underneath surface analysis, a flux of Ar ions (Ar^+^) was used for 3 s for etching the outmost surface layer. The positions of peaks were adjusted using the C 1s binding energy at 284.6 eV. To prepare the samples for FTIR and XPS analyses, ZnS@CPP–AMP colloidal suspensions were poured onto aluminum disks and dried at 40 ± 1 °C until a film was formed.

The morphological and structural characterizations were conducted using transmission electron microscopy (TEM, Tecnai G2-20-FEI, FEI Company, 200 kV). In addition, crystallographic information was obtained by small-area electron diffraction (SAED) patterns. For sample preparation, diluted suspensions (1 : 2, suspension : ethanol) were placed onto a holey-carbon copper grid.

### 2.4. Biological assays

All biological tests were conducted according to the international standard for assessing the toxicity for biomedical applications (Biological evaluation of medical devices: tests for *in vitro* cytotoxicity, International Organization for Standardization, ISO 10993-50). As a proof-of-concept, due to their high lethality in cancer patients, human brain glioblastoma cells (U87-MG) were selected to perform all biological assays *in vitro*. They were procured from the Brazilian Cell Repository (Banco de Células do Rio de Janeiro, BCRJ, Brazil).

The generation of intracellular toxic reactive oxygen species (ROS) was evaluated based on the 2′,7′-dichlorodihydrofluorescein diacetate (DCF-DA) method that measures the fluorescence intensity of 2′,7′-dichlorofluorescein (DCF) species after incubation of ZnS@CPP–AMP with cells.^30^

Oxidative damage occurring in lipids (lipid peroxidation) was assessed based on malondialdehyde (MDA) level using the thiobarbituric acid protocol (TBA test) after 24 h of contact with the nanoconjugate.^30^

The *in vitro* cytotoxicity was estimated by MTT (3-(4,5-dimethylthiazol-2yl-)2,5-diphenyl tetrazolium bromide) assay after immersion with different concentrations of the nanoconjugate with U87-MG cells for 24 h.^3,18,30^

The ZnS@CPP–AMP nanoconjugates were evaluated as fluorescent bioprobes for *in vitro* bioimaging using Eclipse Ti-U (Nikon) epifluorescence microscope.^3,18^

The minimum inhibitory concentration (MIC) test was used to determine the antibacterial activity of εPL solution and ZnS@CPP–AMP nanoconjugates using the agar microdilution method according to the literature.^31,32^

Statistical significance was evaluated using one-way ANOVA (Analysis of Variance) followed by Bonferroni's method.^33^

All specific materials, details, and protocols related to the experimental procedures are also presented in the ESI.†

## 3. Results and discussion

### 3.1. Characterization of ZnS@CPP–AMP nanocolloids

**Fig. 1:**
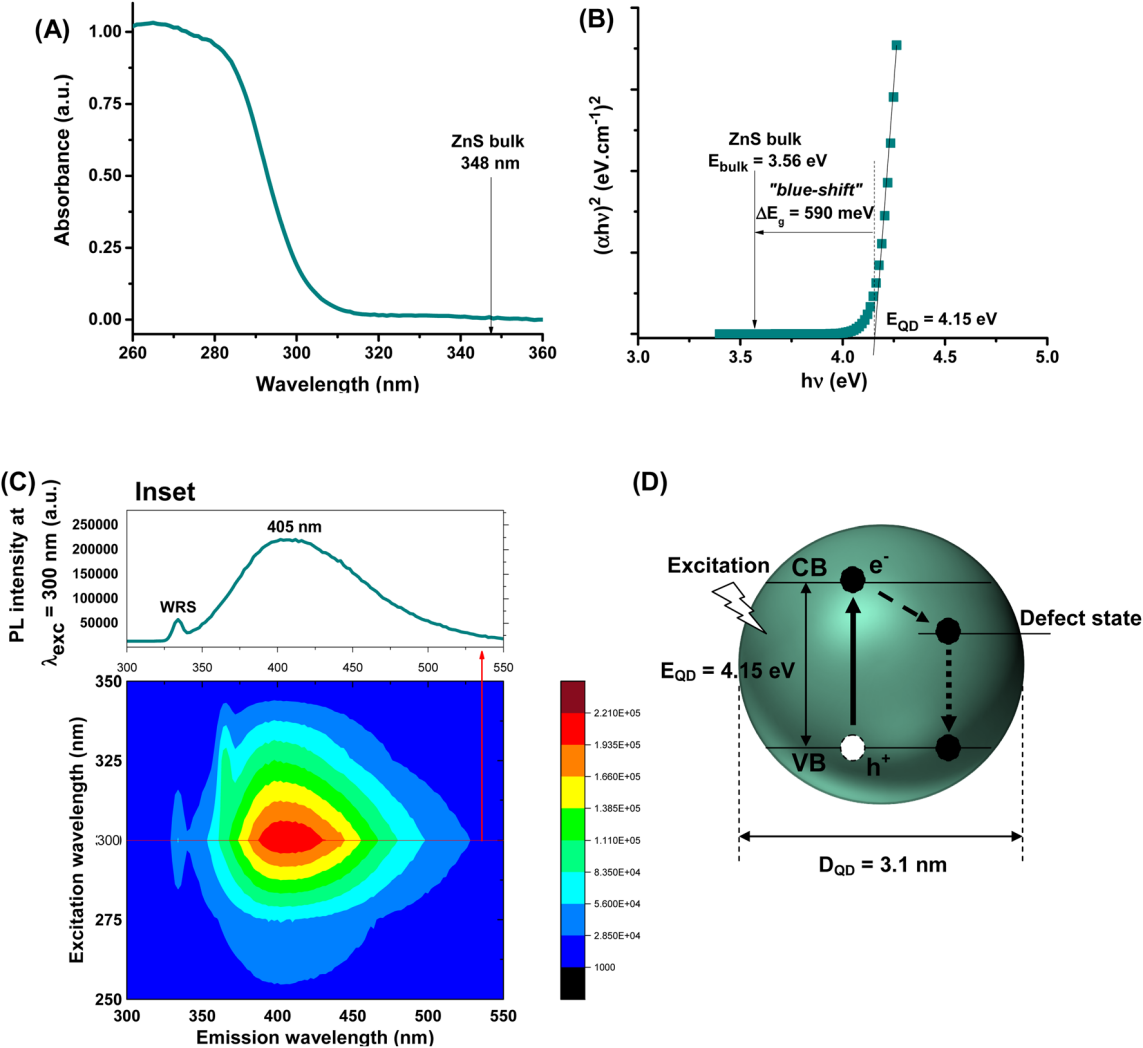
(A) UV-vis absorption spectra, (B) “Tauc relation”, (C) excitation and emission 3D contour plot of ZnS@CPP–AMP conjugates (inset PL spectra at *λ*_exc_ = 300 nm. WRS: Water Raman Scattering). (D) Schematic representation of ZnS QD with information regarding size, band gap structure, and emission pathways based on optical properties. (Not to scale. Continuous line: absorption; dashed line: non-radiative emission; and dotted line: radiative emission. CB: conduction band and VB: valence band; “e^−^”: electron; and “h^+^”: hole.)

Aiming at developing active fluorescent nanoprobes for cancer cell bioimaging, the characterization of optical properties is vital. Hence, the ZnS@CPP–AMP nanoconjugates formed an optically transparent colloidal suspension in the visible range of the light spectrum. Nonetheless, they showed a steep rise in the UV-vis absorption spectrum with a wavelength onset (*λ*_onset_) at approximately *λ*_onset_ = 305 nm (Fig. 1A) associated with the excitonic transition at the ultraviolet region. As a direct wide-bandgap semiconductor, the estimated bandgap energy of ZnS nanocolloids (*E*_QD_) was 4.15 ± 0.10 eV using Tauc analysis^9^ (Fig. 1B). This energy value is larger than the bulk counterpart (*E*_bulk_ = 3.56 eV),^9^ indicating a significant “blue shift” (*i.e.*, shifted to a higher energy; Δ*E*_g_ = *E*_QD_ − *E*_bulk_ = 590 meV). Therefore, it evidenced that ZnS nanoparticles were produced within the “quantum confinement regime”. Considering the semi-empirical model^9^ correlating the average nanoparticle size to the optical bandgap *E*_QD_ (eqn (1)), the ZnS semiconductor core was formed with an average diameter, *D*_QD_ = 3.1 nm, much lower than ZnS Bohr radius (*a*_B_ ∼5.5 nm).^9^ These results demonstrated the effective formation of ZnS quantum dots chemically stabilized and biofunctionalized by the amine-rich polypeptide (*i.e.*, εPL), which behaved as an active surface capping ligand by preventing further nanocrystal growth.

1 Estimated diameter (*D*_QD_) = 2 × {[0.32 − 2.9 × (*E*_QD_ − 3.49)^1/2^]/[2 × (3.50 − *E*_QD_)]}

Regarding the optical emission spectrum of colloidal suspension, the results (inset in Fig. 1C) evidenced the strong photoluminescent activity of the ZnS@CPP–AMP upon excitation (*λ*_exc_ = 300 nm) through a radiative decay, with a maximum signal at *λ*_max_ = 405 nm, in the visible region of the spectrum. The estimated value of full width at half maximum (FWHM) of approximately 100 nm and the relatively large Stokes shift (ΔStokes = *λ*_max_ − *λ*_onset_ = 100 nm) are compatible with the expected behavior of ZnS QDs usually produced by aqueous processes.

Their emissions predominantly result from defect-activated intra-gap states, leading to radiative recombination of charge carriers at energies lower than the excitonic emission.^3,9^ Moreover, 3D excitation–emission plots were performed to evaluate the potential application of ZnS@CPP–AMP as fluorescent nanoprobes for biological imaging. The 3D contour curves (Fig. 1C) indicated that the nanostructure showed a fluorescent “quantum emitter” behavior, with predominant emission within the violet-green window. A summary of ZnS QD features based on optical properties is depicted in Fig. 1D. These findings are very relevant, as they demonstrated the suitability of the ZnS@CPP–AMP nanoconjugates for cell bioimaging, where the approach of “*seeing is believing*” is vital in assisting the earliest possible cancer diagnosis.

**Fig. 2:**
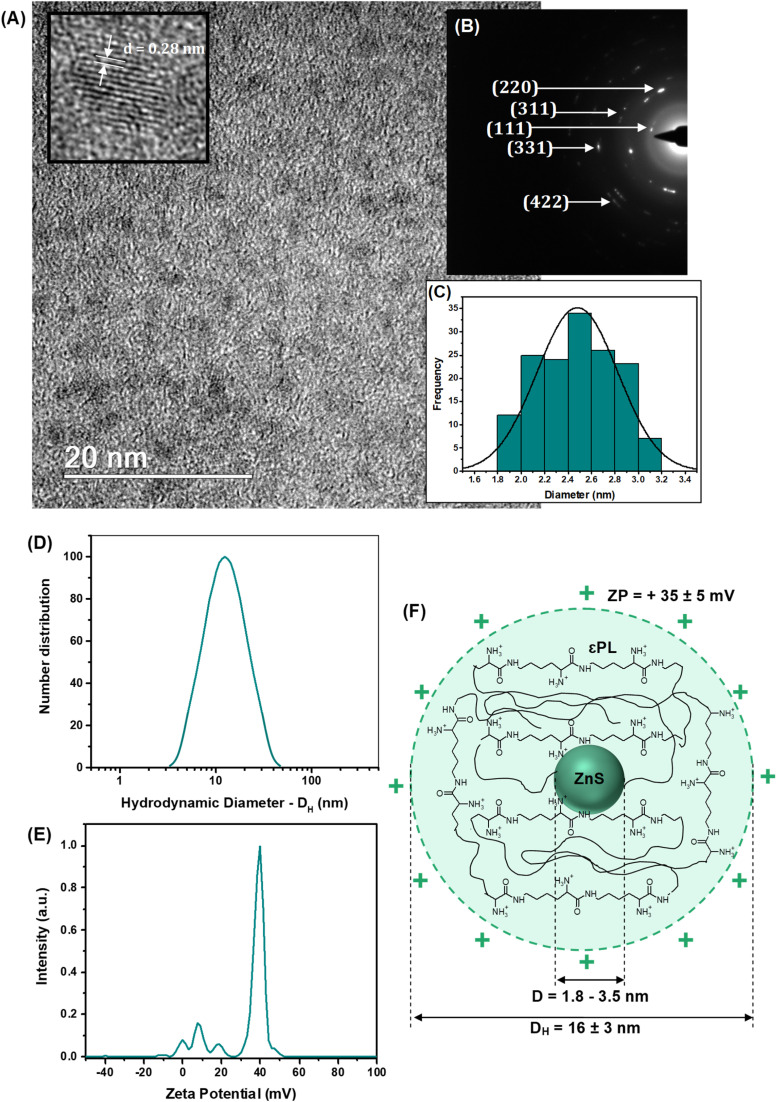
(A) TEM image with HR-image showing lattice fringes, (B) SAED pattern, and (C) histogram of size distribution of ZnS inorganic core. (D) Zeta potential (ZP) and (E) hydrodynamic diameter (*D*_H_) of ZnS@CPP–AMP nanoconjugate. (F) Schematic representation of supramolecular nanostructure of nanoconjugate.

As well-known in nanophotonics, the dimension and structure of semiconductor nanocrystals drastically affect most of their properties, making evaluating size and size distribution a key variable to be addressed. Thus, high-resolution transmission electron microscopy (HR-TEM) images (Fig. 2A) evidenced the formation of fairly uniform and monodispersed spherical nanoparticles with sizes typically ranging from 1.8 to 3.5 nm (average diameter, *D* = 2.5 nm ± 0.3 nm). This average size assessed *via* HR-TEM is consistent with the dimension estimated in the previous section by UV-vis spectroscopy analysis and calculated using a semi-empirical model.^9^

Regarding the crystallinity, the continuous lattice fringes in high-resolution images randomly captured (inset Fig. 2A) confirmed their nanocrystalline structural organization. The estimated interplanar distance (*d* ∼ 0.28 ± 0.05 nm) was assigned to the (111) plane of ZnS, compatible with the “zinc blende” crystalline structure (card #72-2100 – ICCD, International Centre for Diffraction Data). These findings were further endorsed by the selected area electron diffraction patterns depicted in Fig. 2B (rings and spots rings identified).

When bearing in mind the potential application of these nanoconjugates for cell bioimaging, the surface chemistry and colloidal hydrodynamic size in aqueous media play a pivotal role at the biointerfaces, affecting the overall cellular behavior. In particular, the cell endocytosis, the intracellular pathways, and the kinetics of exocytosis of fluorescent nanoprobes are highly affected by the balance of charges at biointerfaces and the colloidal stability of the nanoconjugates. In addition, the surface chemistry of these nanocolloids dictates the antibacterial activity of epsilon-poly-l-lysine (*i.e.*, AMP agent) used as the stabilizing capping ligand. Thus, the results of zeta potential measurements (Fig. 2D, ZP = +35 ± 5 mV) indicated that ZnS@CPP–AMP nanocolloids were positively charged at pH = 5.8 and stabilized principally by electrostatic forces (*i.e.*, ZP > ±30 mV). The positive ZP was credited to the functional amine groups of the εPL polypeptide stabilizing agent of the nanoconjugates, which are protonated below p*K*_a_ (logarithm of acid dissociation constant, *K*_a_) ∼ 10.6.^15^ The acidic pH was chosen to mimic the key characteristic of the tumor microenvironment (TME), which is usually lower than the physiological values of healthy tissues.

The hydrodynamic diameter of the nanoparticles (*D*_H_ = 16 ± 3 nm, Fig. 2E) indicated the formation of water-dispersed colloidal nanostructures, which can be interpreted as the inorganic semiconductor core of ZnS (*D* = 2.5 nm, by TEM) surrounded by solvated εPL polypeptide organic shell layer. The results of ZP and *D*_H_ evidencing the formation of chemically stable colloidal core–shell nanostructures are schematically depicted in Fig. 2F. Considering the cancer nanomedicine applications, these surface charges and hydrodynamic size are suitable for the uptake of the nanoconjugate by tumor cells, which is required for bioimaging and intracellular tracking. At the same time, these findings favor the antibacterial activity of the AMP biomolecule, mostly associated with its positively charged aspect.

**Fig. 3:**
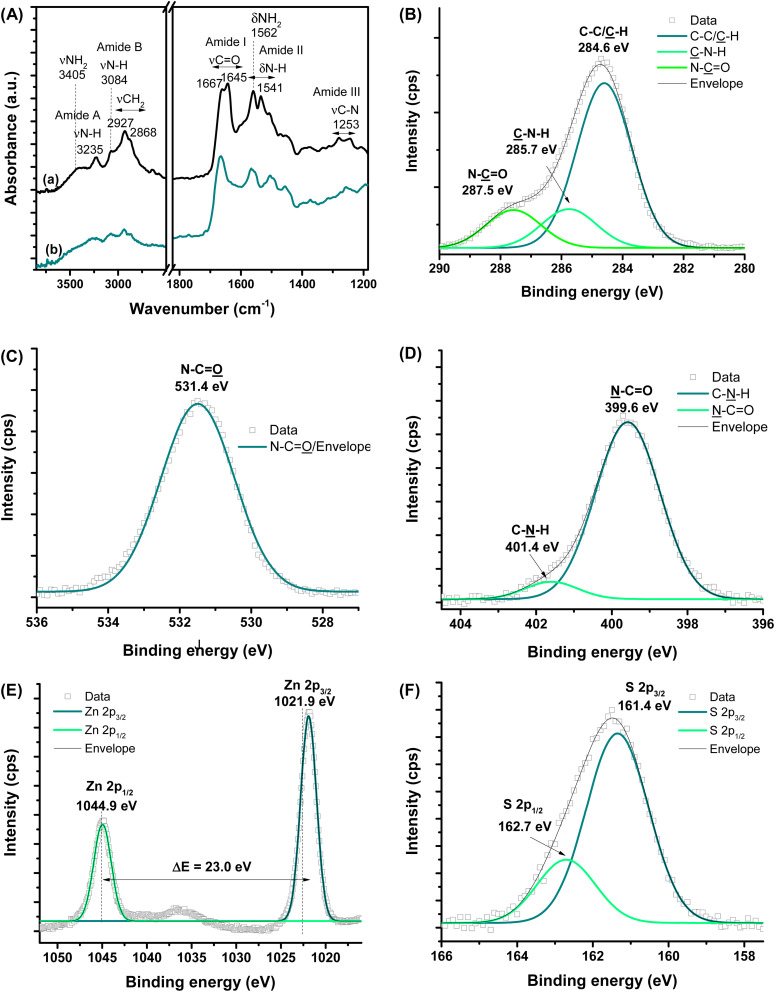
(A) FTIR spectra of (a) εPL polypeptide and (b) ZnS@CPP–AMP nanoconjugate. HR-XPS analysis of (B) C 1s, (C) O 1s, (D) N 1s, (E) Zn 2p, and (F) S 2p regions.

FTIR technique provided a more in-depth investigation of the εPL polypeptide interactions with ZnS QDs at their nanointerfaces (Fig. 3A). The characteristic bands of amine groups (NH_2_/NH_3_^+^) and amide bonds (NH–C=O) of εPL were observed in the pristine material, *i.e.*, before conjugation (Fig. 3A(a) and Table S1†).^34–37^ As a result of the chemical stabilization and functionalization of ZnS QD with εPL, numerous vibrational bands exhibited energy shifts, and changes in their relative intensities were also detected (Fig. 3A(b)). The major vibration modes related to amides, such as amide I at 1690–1600 cm^−1^, amide II at 1575–1480 cm^−1^, and amide III at 1300–1230 cm^−1^, were predominantly affected due to the interactions between the neighboring functional groups. These IR vibrational modes associated with amide bonds are sensitive to the polypeptide chain conformations.^38^ Thus, these FTIR findings confirmed that, after the formation of the nanoconjugates with ZnS QDs, significant changes occurred to the polypeptide chain conformation, which was credited to the interactions between the εPL biopolymer functional groups and the cations at the surface of the nanocrystals (ZnS → εPL). Particularly, the amide I band is sensitive to variations in molecular geometry, and it is helpful for the analysis of the secondary structure and structural changes of proteins and peptides.^38^ Considering the absorbance maximum in the amide I region of spectra, at least two types of secondary structure were observed for εPL peptide and after the ZnS QD synthesis, where the band at 1167 cm^−1^ falls within the β-turn conformation region.^38^

Moreover, the IR spectrum of the biofunctionalized ZnS QDs showed that the prominent band related to protonated amine (–NH_3_^+^) was “blue-shifted” by approximately 6 cm^−1^. In this sense, the differences observed in the FTIR spectra of εPL biomolecule before and after conjugation with ZnS nanocrystals can be interpreted considering the electrostatic interactions mostly driven by the positively charged amino groups (–NH_3_^+^) of εPL peptide and negative anionic sulfide species (S^2−^) at the surface of the inorganic core. In addition, Zn^2+^ ions with dangling bonds at the surface could have established dative bonds with nonbonding electron pairs of N-groups.^39^

Considering the formation of the colloidal ZnS QD (core) capped by the εPL ligand (shell), which predominantly involves superficial and interfacial phenomena, it is important to investigate the nanoconjugate chemistry further using high-resolution XPS spectroscopy analysis (HR-XPS). Hence, at the surface, the different chemical states of C (C–C/C̲–H, C̲–N–H, and N–C̲=O, Fig. 3B), O (N–C=O̲, Fig. 3C), and N (N̲–C=O and C–N̲–H, Fig. 3D) elements, were assigned to the backbone and functional groups of the εPL polypeptide.^40–42^ Upon etching (Ar^+^, 3s), Zn and S species were also observed, which were assigned to the inorganic core of ZnS nanocrystals. The spectra of the Zn 2p region (Fig. 3E) comprise two peaks with the binding energy of 1021.9 ± 0.2 eV (Zn 2p_3/2_) and 1044.9 ± 0.2 eV (Zn 2p_1/2_), with an estimated difference Δ*E* = 23.0 eV, which can be ascribed to the cationic species of Zn^2+^.^3^ The two peaks in the spectrum of the S region (S 2p_3/2_ at 161.4 ± 0.2 eV and S 2p_1/2_ at 162.7 ± 0.2 eV, with Δ*E* = 1.3 eV, Fig. 3F) can be related to sulfur species, present in metal–sulfides (M–S).^3^

### 3.2. Biological assays

#### 3.2.1. Assessment of cytotoxicity

ZnS quantum dots have emerged as promising non-toxic alternatives to highly hazardous QDs based on Cd and Pb, although some concerns still remain regarding their biosafety, as for other nanosized materials, for biomedical and nanomedicine applications. Reactive oxygen species (ROS) production is the underlying reason for eliciting nanomaterial toxicity in cells. ROS is a common byproduct of cellular metabolic reactions and, at moderate levels, performs several roles in physiological processes. Conversely, higher ROS levels can lead to an oxidative/antioxidative system imbalance, called oxidative stress, resulting in irreparable damage to cells and tissues.^22,30,43^ To address this concern, intracellular ROS formation induced by ZnS@CPP–AMP was evaluated using a fluorescent probe (DCFH-DA), and the assessment of oxidative damage in lipids, which is one of the major components of the cell membrane, was based on malondialdehyde, a byproduct of lipid peroxidation (LiP).^22,30,43^

**Fig. 4:**
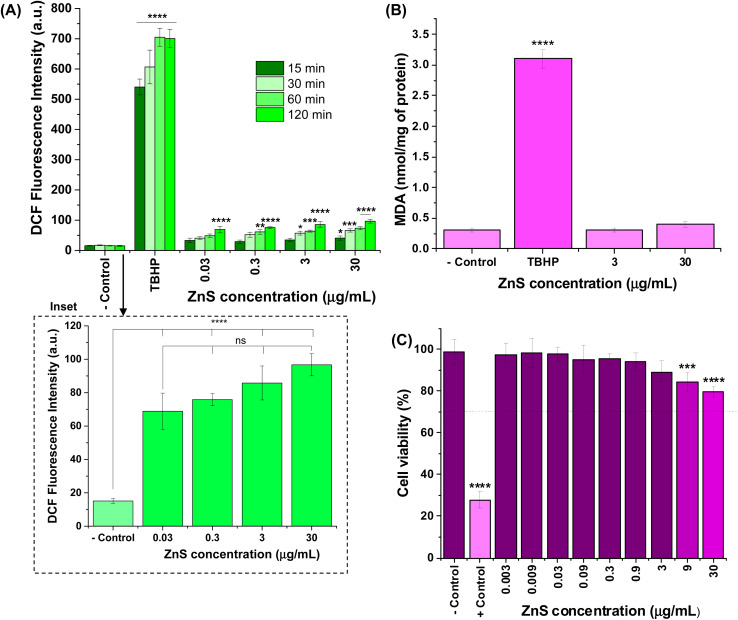
(A) PL intensity associated with accumulation of intracellular ROS in U87-MG cells induced by different concentrations of ZnS@CPP–AMP after 15, 30, 60, and 120 min of exposure (inset: detail of effect of concentration at 120 min). (B) Results of lipid peroxidation assessed by malondialdehyde levels. (C) Cytotoxicity of ZnS@CPP–AMP at different concentrations after incubation for 24 h with U87-MG cell line. Statistical analyses of ‘one-way’ ANOVA, Bonferroni, and multiple comparison test: **** = *p*-value, *p* < 0.0001, ** = *p* < 0.01, and * = *p* < 0.05. Only the comparisons to the respective “− control” were presented in graphs, except for the inset, where the non-significant (ns) differences between samples were also indicated.

The results of intracellular ROS production were expressed in fluorescence intensity of 2′,7′-dichlorofluorescein species, which were formed *in vitro* after internalization and hydrolysis of DCF-DA in the presence of H_2_O_2_, and the radicals hydroxyls (OH˙), peroxyls (ROO˙) inside the cells (Fig. 4A). As a general trend, a very low increase in the relative intensities of intracellular ROS was observed compared to “− control” (cells treated with DCF-DA without nanoconjugates) after exposure to ZnS@CPP–AMP up to 120 min. Considering four tested concentrations of colloidal nanoconjugates (0.03, 0.3, 3, and 30 μg mL^−1^), the fluorescence values after 120 min of incubation were the only ones statistically different from PL intensity measured at 15 min. Moreover, the ROS formation was not dependent on ZnS@CPP–AMP concentration, which means that no detectable changes in the amount of these reactive species were observed by increasing its concentration by up to 1000 times (for example, see inset in Fig. 4A for 120 min). All the samples differed statistically (ANOVA-one way, Bonferroni teste, significance level *α* = 0.05) from cells exposed to the positive control (*tert*-butyl hydroperoxide, TBHP). So, these results demonstrated that ZnS@CPP–AMP nanoconjugates induced no relevant intracellular ROS, which supports the well-accepted non-toxicity and biosafety of ZnS-bearing nanomaterials.

Additionally, intracellular lipid damage was evaluated by the amount of malondialdehyde formed, and it was assessed by using the thiobarbituric acid protocol as one indicator of oxidative stress.^30,43^ The results shown in Fig. 4B indicated that no statistically significant increase in MDA was found in the cells exposed to ZnS@CPP–AMP at the concentrations of 3 and 30 μg mL^−1^ when compared to the negative control (ANOVA-one way, Bonferroni test, *α* = 0.05). These findings corroborated that the ZnS@CPP–AMP nanoconjugates promoted no significant oxidative species generation after cellular uptake.

As an additional supporting tool for the ROS tests to confirm the absence of cytotoxicity of ZnS@CPP–AMP nanoconjugates, MTT assays were conducted based on the international standard (Part 5: Tests For *In vitro* Cytotoxicity: Biological Evaluation of Medical Devices – ISO 10933-5). After incubation of the samples with U87-MG cancer cells for 24 h, the number of viable cells depended on ZnS@CPP–AMP colloid concentration (Fig. 4C). Even at the highest tested concentration (30 μg mL^−1^) of nanoconjugates, the cell viability response (>75%) indicated no cytotoxic effect *in vitro*. So, relying on these ROS assays and cell viability results, using U87-MG cancer cells as *in vitro* models, the ZnS@CPP–AMP nanoconjugates were demonstrated to be non-toxic and biosafe for cellular bioimaging as suitable fluorescent nanoprobes.

#### 3.2.2. Cellular uptake and bioimaging of ZnS@CPP–AMP

**Fig. 5:**
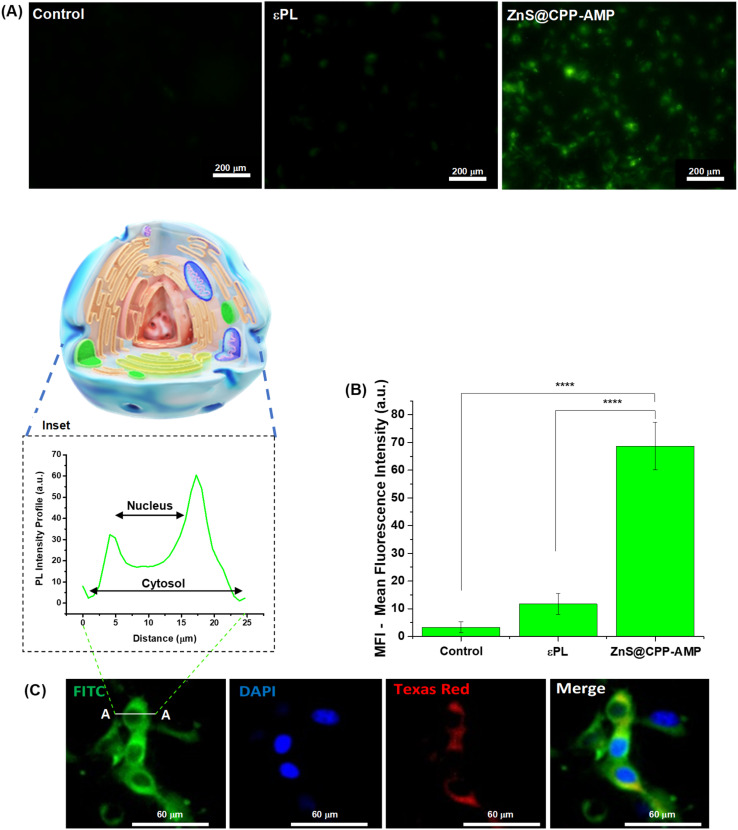
*In vitro* labeling of GBM cancer cells (U87-MG) with ZnS@CPP–AMP in comparison to cells without treatment (control) and incubated with εPL for 30 min: (A) Fluorescence images (FITC, fluorescein isothiocyanate, filter, scale bar = 200 μm) and (B) fluorescence intensity profiles. (C) Fluorescence images showing (FITC) green emission of ZnS@CPP–AMP (inset: fluorescence intensity profile along line A–A); (DAPI) DNA staining with 4′,6-diamidino-2-phenylindole solution in blue color (nuclei biomarker); (Texas red, sulforhodamine 101 acid chloride) red emission of LysoTracker^®^ (lysosomal biomarker); and (merge) overlapped images of green, blue, and red emissions for U87-MG cells after 30 min of treatment (scale bar = 60 μm). Statistical analyses of ‘one-way’ ANOVA, Bonferroni, and multiple comparisons: **** = *p* < 0.0001 (*n* = 3).

The cellular uptake of ZnS@CPP–AMP was investigated in glioblastoma cancer cells, relying on the photoluminescent properties of this nanoconjugate, as evaluated in Section 3.2.1. The fluorescence microscopy results for U-87 MG cells incubated with ZnS@CPP–AMP revealed strong green fluorescence emission from QDs after internalization (Fig. 5A). As controls, no significant fluorescence was detected for glioblastoma cells without exposure to the nanoconjugates and after contact with εPL solution (*i.e.*, only the polypeptide, no ZnS QD), demonstrating that the ZnS inorganic semiconductor core was responsible for the fluorescence. The Mean Fluorescence Intensity (MFI) of cells (Fig. 5B) from the images was performed with NIS-Elements software (Nikon Instruments, USA) and indicated a significant statistical difference (ANOVA-one way, Bonferroni teste, *α* = 0.05) between the QD samples and controls. For intracellular transport, the cell membrane usually promotes the engulfment of molecules and extracellular fluid in an intracellular vesicle by invagination that will subsequently traffic through the cell along the endosomal–lysosomal pathway.^9,18^ Thus, the LysoTracker®, a deep-red biomarker, was applied to stain late endosomes and lysosomes (acid organelles), tracking nanoconjugate uptake *via* the endocytosis pathway. After 30 min of contact with U87-MG cancer cells, the ZnS@CPP–AMP nanoconjugates depicted good co-localization with the late endosomes/lysosomes of cells, as demonstrated by the yellow regions in the cytosol. These regions were identified by overlapping the green fluorescence from QDs and the red fluorescence from the LysoTracker® probe (Fig. 5C). Staining with DAPI (4′,6-diamidino-2-phenylindole, DNA biomarker) was applied to target and visualize the cell nucleus. In these images, as displayed in Fig. 5C (inset), it could be clearly seen that the spatial distribution of ZnS@CPP–AMP nanoconjugates within the cells. The green fluorescent signal was uniformly scattered in the cellular cytosol with no evidence of significative emission localized in the region associated with the cell nucleus.

This response was expected because QDs are entrapped inside the cell cytosol vesicles, as the delivery of freely diffusing QD probes into the cytoplasm of cells usually needs additional strategies not exploited in this research.

The paramount importance of the earliest possible detection of highly lethal types of cancer was precisely stated in a recent viewpoint report by Bhatia and co-workers,^44^ as quoted: “*An essential challenge in cancer detection is diagnosing lethal cancer (that is, not cancer one may die with but cancer one will die from) at earlier stages when intervention can have the greatest impact on outcomes*.”

These results demonstrated that ZnS@CPP–AMP nanoconjugates developed in this study could be potentially used as fluorescent probes for cell bioimaging, where GBM cells, the most lethal form of brain tumors, were successfully used as a proof-of-concept. Thus, based on these promising findings associated with future research integrating cancer cell targeting features, these fluorescent nanoconjugates can aid in detecting, diagnosing, and monitoring cancer progression and fluorescence guidance for intraoperative tumor identification.

#### 3.2.3. Antibacterial activity of the nanostructures

One of the novelties developed in this study regards using εPL polypeptide as the ligand for the chemical stabilization of ZnS QDs nanocolloids in an aqueous medium combined with its biofunctionalization as an antimicrobial peptide. Considering the potential application of these nanoconjugates in oncology, cancer patients are often immunosuppressed by chemotherapy, offering opportunist pathogenic infections. Thus, the antibacterial activities of εPL solution and ZnS@CPP–AMP nanoconjugates were assessed based on the lowest antimicrobial agent concentration (MIC) that could inhibit the growth of the tested strains using the microdilution method. The MIC results against Gram-negative (*E. coli*) and Gram-positive (*S. aureus*) reference strains were summarized in Table 1. MIC values of pure εPL solution (*i.e.*, the reference antibacterial polypeptide) were 300 μg mL^−1^ for both tested strains. Moreover, the antibacterial activity of ZnS@CPP–AMP nanoconjugate was relatively higher than that observed for the εPL solution for the reference bacteria strains.

**Table 1:** Minimum inhibitory concentration results

Minimum inhibitory concentration tests (μg mL−^1^)			
Antimicrobial	MIC range	Reference strains	
		*S. aureus* ATCC^b^ 29213	*E. coli* ATCC^b^ 25922
**εPL solution** a	**0.58–300**	**300**	**300**
**ZnS@CPP–AMP** a	**0.58–300**	**9.37**	**37.5**
Ciprofloxacin	0.12–64	0.5	<0.12
Gentamicin	0.12–64	1	1
Tetracycline	0.12–64	0.12	1

a Concentration of εPL polypeptide in solution or forming the nanoconjugate.

b American Type Culture Collection.

Thus, these results confirmed another hypothesis posed in this research, where the εPL, used as a biofunctional capping ligand for nucleation and growth of colloidal ZnS QDs, demonstrated antibacterial activity. No relevant antibacterial effect from the ZnS inorganic core was expected based on the analogy with the previous ROS assays using cells. So, besides the cationic interactions expected with predominantly anionic bacterial biomembranes, the higher activity of εPL after the formation of the nanoconjugate (ZnS@CPP–AMP) can be credited to changes in its three-dimensional conformation, which affected the interactions with biomembrane-associated molecules.^45^

## 4. Conclusions

In summary, this research demonstrated that new ZnS QDs directly stabilized by epsilon-poly-l-lysine (εPL) were effectively synthesized using an eco-friendly aqueous route, leading to the formation of supramolecular colloidal nanoconjugates (ZnS@CPP–AMP). They displayed water-dispersed core–shell positively charged nanostructures (*D*_H_ = 16 ± 3 nm, ZP = + 35 ± 5 mV). The inorganic core composed of ZnS semiconductor nanocrystals showed a uniform spherical-like shape associated with a narrow size distribution (*D* = 2.5 ± 0.3 nm). These nanohybrids were demonstrated to be non-cytotoxic based on the MTT cell viability responses and intracellular generation of ROS. More importantly, brain cancer cells successfully internalized the ZnS@CPP–AMP nanocolloids, proving to be luminescent nanoprobes for bioimaging cells *in vitro*. Moreover, due to the presence of the εPL as a biofunctional cationic molecule, these nanohybrids demonstrated antibacterial activity against Gram-positive and Gram-negative bacteria, including drug-resistance field strains. Thus, the positive net surface charge combined with the very low colloidal hydrodynamic dimension of bioconjugates have favored the tumor-cell uptake as well as the antibacterial activity. Therefore, it can be envisioned that these innovative fluorescent colloidal nanohybrids offer an avenue of alternatives of multifunctional and sustainable nanomaterials that may be associated with specific affinity biomolecules for targeted bioimaging cancer cells and inactivating pathogenic bacteria.

## Conflicts of interest

All authors have read the manuscript, understood the content, and agreed to the published version. The authors confirm no competing interests to declare regarding the publication of this article.

## Supplementary Material

RA-013-D3RA06711D-s001
