# Supplementary material for: Bio-functionalized nanocolloids of ZnS quantum dot/amine-rich polypeptides for bioimaging cancer cells with antibacterial activity: “seeing is believing”
Source: RSC Adv. 2023 Nov 23;13(49):34378–90. doi: 10.1039/d3ra06711d (PMC10665648; doi:10.1039/d3ra06711d)
Supplement: RA-013-D3RA06711D-s001 [file RA-013-D3RA06711D-s001.pdf]

Electronic Supplementary Material (ESI) for RSC Advances.
This journal is © The Royal Society of Chemistry 2023

Electronic Supplementary Material

Bio-Functionalized Nanocolloids of ZnS Quantum dot/amine-rich Polypeptides for Bioimaging Cancer Cells with Antibacterial Activity: *"Seeing is Believing"*

Alexandra A. P. Mansur[1], Sandhra C. Carvalho[1], Elaine M.S. Dorneles[2], Andrey P. Lage[3], Zelia I. P. Lobato[3], Herman S. Mansur[1,*]

[1]*Department of Metallurgical and Materials Engineering, Center of Nanoscience, Nanotechnology, and Innovation-CeNano²I, Federal University of Minas Gerais, UFMG, Brazil;*
[2]*Departamento de Medicina Veterinária, Universidade Federal de Lavras, UFLA, Brazil;*
[3]*Departamento de Medicina Veterinária Preventiva, Federal University of Minas Gerais, UFMG, Brazil;*
*e-mail: *hmansur@demet.ufmg.br*

Materials and Methods

. Cell culture

U87-MG (passage 44) cells were cultured in DMEM (Dulbecco's Modified Eagle Medium, Gibco, USA) with 10% FBS (fetal bovine serum, Cripion Biotecnologia Ltda., Brazil), penicillin G sodium (10 units/mL) + streptomycin sulfate (10 mg/mL) + amphotericin-b (0.025 mg/mL) (Antibiotic-Antimycotic, Gibco, USA) in a atmosphere of 5% CO_2 at 37 ˚C.

. Measurement of intracellular ROS

U87-MG cells were plated at 1×10^4 cells/well on 96-well microplates. The cell population was synchronized by nutrient deprivation for 24 h (DMEM medium without FBS, 37 ˚C/5 % CO_2). Then, cells were incubated with 100 µL of 2',7'-dichlorodihydrofluorescein diacetate solution (DCF-DA, 100 µM diluted in DMEM medium, Sigma-Aldrich, USA) for 40 min at 37 ˚C/5 % CO_2. After this period, the probe was removed, and U87-MG cells were exposed to 100 µL of ZnS@CPP-AMP nanoconjugates at 0.03, 0.3, 3, and 30 µg/mL (related to ZnS inorganic core concentration). Cells were also incubated with tert-butyl hydrogen peroxide (TBHP, 5.0 µM in water, Sigma-Aldrich, USA), as "+ control", and the cells only treated with DCF-DA were the "- control". After incubation times of 15 min; 30 min; 60 min; and 120 min, at 37 ˚C/5 % CO_2, the green fluorescence emission of DCF was measured using Varioskan™ LUX multimode microplate reader (Thermo Scientific, $\lambda_{excitation}$ = 488 nm, and $\lambda_{emission}$ = 528 nm). Data were presented as the mean and standard deviation of six replicates (n = 6).

. Determination of lipid peroxidation

* To whom correspondence should be addressed: Federal University of Minas Gerais, Av. Antônio Carlos, 6627 – Escola de Engenharia, Bloco 2 – Sala 2233, 31.270-901, Belo Horizonte/MG, Brazil; Tel/Fax: +55-31-34091843; E-mail: hmansur@demet.ufmg.br (H. Mansur)

U87-MG cells were plated (2×10^4 cells/well, 6-well plates) and synchronized for 24 h. In the sequence, nanoconjugate suspension was added to wells at the final ZnS concentration of 3 and 30 μg/mL. Positive control ("+ control") was U87-MG cells with DMEM, 10% FBS, and 5 μM of TBHP. Negative control ("- control") was U87-MG cells with DMEM, 10% FBS and chips of sterile polypropylene Eppendorf®, 1 mg/mL). After 24 h incubation, cells were washed two times by Tris–HCl buffer (400 mM, pH 7.3, Labsyntth Produtos para Labotatórios Ltda, Brazil) and treated with 1 mL of a solution containing 0.4% (w/v) thiobarbituric acid (TBA, Sigma-Aldrich, USA), 0.5% (w/v) sodium dodecyl sulfate (SDS, LCG-Biotecnologia, Brazil), and 5% (v/v) acetic acid (Labsyntth Produtos para Labotatórios Ltda, Brazil) at final pH of 3.5 ± 0.1. Next, the cells were scraped and incubated at 95 ˚C (Termomix, Eppendorf F1.5). After 60 min, the reaction was stopped in the ice bath for 5 min. Then, a volume of 300 μL was transferred to a 96-well plate, and the final pink-colored solution formed from the reaction between thiobarbituric acid and malondialdehyde (MDA) was subjected to absorbance analysis (Varioskan™ LUX multimode microplate reader, Thermo Scientific) at λ = 532 nm. The results were calculated as nmol of MDA-TBA/mg of cellular protein using 156 $mM^{-1}cm^{-1}$ as a molar extinction coefficient of MDA-TBA. Proteins extracted from cells were calculated using the Bradford method (Bradford reagent, Sigma-Aldrich, USA) using bovine serum albumin (BSA, Sigma-Aldrich, USA) as reference material. Data were presented as the mean and standard deviation of three replicates (n = 3).

. Cytotoxicity protocol

Cells were plated (1×10^5 cells/well, 96-well plates) and synchronized for 24 h. After that, ZnS@CPP-AMP nanoconjugate suspension was added to individual wells at different final concentrations per well (related to ZnS inorganic core concentration) as follows: 0.003, 0.009, 0.03, 0.09, 0.3, 0.9, 3, 9, and 30 μg/mL. Positive control ("+ control") was cells cultured with DMEM, 10% FBS, and 1.0% v/v Triton™ X-100 (Sigma-Aldrich, USA). Negative control ("-control") was cell cultured with DMEM, 10% FBS and chips of sterile polypropylene Eppendorf®, 1 mg/mL (Eppendorf, Germany). After 24 h, the total volume of the solution of each well was aspirated and replaced with culture media containing serum (60 μL) in each well. Then, the (3-(4,5-Dimethylthiazol-2yl)2,5-diphenyl tetrazolium bromide reagent (MTT, 5 mg/mL, Sigma-Aldrich, USA) was added to each well and incubated at 37°C/4 h/5% CO_2. Next, 40 μL SDS solution/4 % HCl (37 %, Sigma-Aldrich, USA) was placed in each well and incubated at 37 ˚C/16 h/5% CO_2. Then, the volume of 100 μL from each well was aspirated and transferred to a blank 96-well plate, and the absorbance was evaluated using iMark™ Microplate Absorbance Reader (Bio-Rad®, USA) at λ = 595 nm. The percentage of cell viability was estimated according to Eq. S1 after blank corrections, and the values of the control group

were set to 100% of cell viability. Data were presented as the mean and SD of six replicates (n = 6).

$$Cell\ viability\ =\ \frac{Absorbance\ of\ sample\ and\ cells}{Absorbance\ of\ control}\ \mathrm{x}\ 100 \qquad \text{(S1)}$$

. Cell uptake of nanoconjugates - Bioimaging

U87-MG cells were plated (5×10^5 cells/well, 6-well plate) and synchronized for 24 h. Then, samples (1:1, v/v, ZnS@CPP-AMP:DMEM with 10 % FBS) were added to the cell and incubated at 37 ˚C /5 % CO_2 for 30 min, then washed with PBS solution.

Then, the U87-MG cells were fixed with paraformaldehyde (Sigma-Aldrich, USA) solution (4.0 % in phosphate-buffered saline, PBS, Gibco, USA) for 30 min, washed three times with PBS, and coverslips were mounted with Hydromount® (Thermo Fisher Scientific Ltd., USA). Digital images were captured in the sequence with a Ti-U epifluorescence microscope (Nikon Instruments, USA) using a FITC filter cube for ZnS green emission. To reveal late endosome/lysosome vesicles and cell nucleus, U87-MG cells after 30 min of incubation with ZnS@CPP-AMP nanoconjugate were washed and additionally stained with LysoTracker® Deep Red (Thermo Fisher Scientific, USA) and DAPI (4′,6-diamidino-2-phenylindole, Thermo Fisher Scientific, USA), respectively, according to the manufacturer's protocol. For blue fluorescence emission, a DAPI filter cube was used. Images were acquired using a Texas Red filter cube for deep red-emission detection. Three-color confocal fluorescence images were recorded separately in the correspondent channel and merged afterward.

. Antibacterial Activity

To determine the antibacterial activity of εPL solution and ZnS@CPP-AMP nanoconjugates, the minimum inhibitory concentration (MIC) was used using the agar microdilution method. For the MIC protocol, from an initial concentration of the treatment (εPL = 300 μg/mL for both εPL solution and ZnS@CPP-AMP), several concentrations were tested using a 2-fold dilution to determine the lowest concentration that inhibits the growth of a strain of bacteria. Samples were tested against the reference strains *Escherichia coli* (ATCC 25922) and *Staphylococcus aureus* (ATCC 29213). The antibiotic standards (ciprofloxacin, gentamycin, and tetracycline, Sigma-Aldrich, USA) were also tested against the reference strains to ensure that the results were within acceptable quality control limits for susceptibility testing according to Clinical and Laboratory Standards Institute (CLSI) guidelines [1].

Results and discussions

Table S1 - FTIR bands of εPL polypeptide [2-5].

Band assignment	Wavenumber (cm^{-1})
Stretching of $-NH_2$	3405
Stretching of Amide A (N-H)	3235
Stretching of Amide B (N-H)	3084
Stretching of $-CH_2$	2927 and 2868
Stretching of protonated Amide I (C=O) - $CH(NH_3^+)$-C(=O)-NH	1667
Stretching of deprotonated Amide I (C=O) - $CH(NH_2)$-C(=O)-NH	1645
Bending of $-NH_2$	1562
Bending of Amide II (N-H)	1541
Stretching of Amide III (C-N)	1253

References

1. Clinical Laboratory Standards Institute - CLSI. Performance Standards for Antimicrobial Susceptibility Testing, 27th Edition. Clinical and Laboratory Standards Institute, Wayne, M100, 2017. 249p.

2. Lv, J. -M., Meng, Y. -C., Shi, Y. -G., Li, Y. -H. Chen, J., Sheng, F. Food Process. Preserv., 44, 2020, e14320.

3. Wang, R., Zhou, B., Xu, D. -l., Liang, H. X. L., Feng, X. -h., Ouyang, P. -k., et al.. J. Bioact. Compat. Polym., 31, 2016, 242 –259.

4. Maeda, S., Fujiwara, Y., Sasaki, C., Kunimoto, K. -K.. Polym. J., 44, 2012, 200–203.

5. Maeda, S., Kunimoto, K. -K., Sasaki, C., Kuwae, A., Hanai, K. J. Mol. Struct., 655, 2003, 149–155.
